# Green synthesis of silver nanoparticles with *Torenia fournieri* leaf extracts and assessing the antioxidant and antibacterial properties, *para*-nitrophenol catalysis, and nanotoxicity

**DOI:** 10.1039/d5ra10074g

**Published:** 2026-03-17

**Authors:** Mathivathani Kandiah, Rahma Arifeen, Beneli Gunaratne, Ominda Perera

**Affiliations:** a Faculty of Life and Medical Sciences Business Management School (BMS) Campus Colombo 00600 Sri Lanka mathi@bms.ac.lk

## Abstract

Nanotechnology provides new opportunities in medicine and environmental remediation through the development of metal-based nanoparticles. This study focused on the green synthesis of silver nanoparticles (AgNPs) using five varieties of *Torenia fournieri*; Deep blue, Blue White, Rose, Burgundy, and Lemondrop. The purpose was to evaluate the potential of plant-based AgNPs in catalytic, antimicrobial, antioxidant, and cytotoxic applications. The optimal conditions for AgNP synthesis were 90 °C for 60 minutes. Scanning Electron Microscopy revealed spherical 25–60 nm Lemondrop_AgNPs. Antioxidant profiling indicated that AgNPs had higher Total Phenolic Content, Total Flavonoid Content, and Total Antioxidant Capacity compared to water extracts (WEs). *Para*-Nitrophenol catalysis was observed at 4000 ppm AgNPs within 45 minutes, with Deepblue_AgNPs showing the highest rate constant (*k* = 0.0211 min^−1^). All AgNPs showed higher antibacterial activity against *Staphylococcus aureus* and *Escherichia coli* compared to WEs, with *Escherichia coli* showing higher zones of inhibition. Cytotoxicity of AgNPs using *Artemia salina* was assessed, and 100% viability was recorded at 200 ppm and 800 ppm of AgNPs. *In silico* docking studies revealed that the 3-atom Ag cluster showed lower binding energy (−1.11 kcal mol^−1^) than the Ag atom (−0.19 kcal mol^−1^) when docked to protein Mre-11. The findings indicate that *Torenia*-mediated AgNPs are safe and possess multifunctional properties suitable for bioremediation, wastewater treatment, and pharmaceutical applications. This study provides the first report of AgNP synthesis using five varieties of *Torenia fournieri*, highlighting the originality of this plant-based approach and its promise for sustainable nanotechnology.

## Abstract 2

Nanotechnology provides new opportunities in medicine and environmental remediation through the development of metal-based nanoparticles.

## Graphical abstract

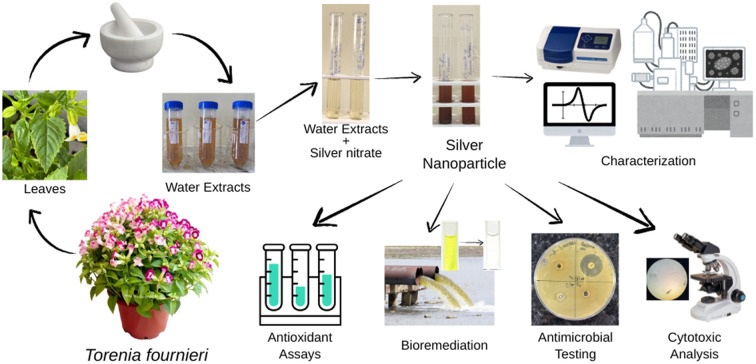

## 1 Introduction

Plant-based silver nanoparticle (AgNP) synthesis has garnered considerable attention due to its wide range of applications in catalytic activity, water purification, and biomedical science, as it is simple, cost-effective, and eco-friendly.^1^ Plants are abundant, less complicated than microbial methods of AgNP synthesis, and contain many bioactive compounds (phytochemicals) that function as stabilising and reducing agents, which can effectively reduce metal ions to form nanoparticles. This method not only eliminates the need for toxic chemicals but also provides nanoparticles with enhanced stability and bio-functionalization capabilities.^2^

In tropical and subtropical regions of Asia, Africa, and Madagascar, *Torenia fournieri* (*T. fournieri*) is a commonly cultivated ornamental plant.^3^ It is known for its diuretic, detoxifying, and anti-inflammatory properties and has been used to cure many human ailments, such as gastroenteritis, hepatitis, hypertension, and pneumonia.^4^ Its potential as an anticancer agent has been highlighted in recent studies, as *Torenia* sp. contains bioactive compounds such as flavonoids and phenols, which are known to exhibit strong antioxidant properties. These compounds scavenge reactive oxygen species, highly reactive molecules that cause cellular damage, ageing, cancer progression and other oxidative stress-related diseases. This makes *Torenia* sp. an excellent source for nanoparticle synthesis equipped with enhanced therapeutic properties.^5^

Nanotechnology has attracted significant attention in both medical and environmental research due to its unique size-dependent properties and a high surface-to-volume ratio.^6^ Its ability to manipulate materials at the molecular level has paved the way for innovations in drug delivery systems, diagnostics, and therapeutic agents, with nanoparticles emerging as strong candidates in cancer therapy, antimicrobial treatment, and the reduction of oxidative stress.^7^ Among nanoparticles, metal nanoparticles have gained significant importance due to their enhanced properties compared to bulk metals.^8^ AgNPs, in particular, have been extensively studied due to their antimicrobial activity, unique optical properties, and biocompatibility.^9^ Silver has long been recognised for its medicinal benefits in wound healing and antimicrobial treatment, and synthesising it at the nanoscale further enhances its effectiveness.^10^ AgNP synthesis can be achieved through two major strategies: top-down and bottom-up. The top-down method involves mechanically or chemically breaking bulk material into nanosized particles, while the bottom-up method assembles smaller atomic or molecular units into nanoparticles.^11^ In green synthesis of metal nanoparticles, this bottom-up approach is driven by bioactive compounds that reduce and stabilise the metal ions to form respective nanoparticles.^12^

Conventional methods of AgNP synthesis, such as chemical reduction, microwave-assisted synthesis, pyrolysis, ball-milling, and laser ablation, are effective but costly, environmentally damaging, and dependent on toxic reagents.^13^ Furthermore, chemically synthesised nanoparticles have been shown to induce harmful effects.^14^ As a result, interest has shifted toward eco-friendly, biosynthetic methods that use biological resources such as microbial and plant extracts.^15^ Although microorganisms are good candidates for the bio-fabrication of nanoparticles, the process is complicated, there is a risk of contamination in the culture medium, and the size of the nanoparticles cannot be controlled. Unlike microbes, plants do not require genetic modification, and the synthesis process is less toxic. Overall, plant-based methods are preferred over microbial synthesis and offer a more sustainable, cost-effective alternative, especially in large-scale applications.^16^

Despite these advantages, plant-mediated synthesis poses certain limitations, including challenges associated with large-scale production and a limited shelf life due to the incorporation of biological capping agents that degrade with time. Further, the use of different plant species and extraction methods may affect reproducibility and produce fewer nanoparticles, as plants produce fewer secreted proteins. However, these limitations can be overcome through additional surface functionalisation, the addition of stabilising agents to enhance nanoparticle stability and shelf life, the selection of plant species that are readily available and suitable for large-scale processing, and the careful optimisation of synthesis parameters.^17,18^

AgNPs have attracted considerable interest in bioremediation applications due to their catalytic properties, particularly for the reduction of *p*-nitrophenol (PNP), a hazardous industrial compound, to *para*-aminophenol (PAP).^19^ Compared with conventional methods, AgNPs can effectively degrade these harmful compounds into less toxic products, making them a promising solution for addressing water pollution. This helps protect water bodies and reduce the environmental damage caused by industrial waste.^20^

Beyond these uses, AgNPs are well known for their antimicrobial activity, particularly in the context of growing antimicrobial resistance. They exhibit strong non-specific toxicity against both Gram-negative and Gram-positive bacteria by disrupting cell membranes and interfering with essential cellular processes.^21^ Further, photocatalytic nanomaterials, including halide perovskite nanoparticles, have emerged as promising next-generation antibacterial agents due to their ability to generate reactive oxygen species under light irradiation, offering a controllable and efficient approach to bacterial inactivation.^22,23^

Additionally, AgNPs have demonstrated cytotoxic properties in tumour therapy.^24^ Computational approaches, such as docking studies, allow researchers to investigate the interaction of Ag atoms with proteins associated with cancer progression. These studies provide valuable insights into the molecular mechanisms by which AgNPs may exert their anticancer effects, thereby allowing the design of more effective and targeted cancer therapies.^25^ Among the proteins involved in tumour survival is the MRN (MRE11–RAD50–NBS1) complex, which detects and repairs DNA double-strand breaks through homologous recombination and non-homologous end joining.^26^ MRE11 protein repairs DNA through its nuclease activity. Mutations in MRE11 are seen in breast, colorectal, and *BRCA*-mutant tumours.^27^ Therapeutically, the MRN complex has gained attention as a target in cancers with DNA repair deficiencies. Most efforts have focused on inhibiting the nuclease activity of MRE11.^28^ Docking studies have shown that Ag atoms binding to the NBS1-interaction site on MRE11 can impede MRN assembly, thereby compromising double-strand break repair in tumour cells.^29^

While AgNPs are usually considered safe and non-toxic, researchers are particularly concerned about their behaviour in the environment and their toxic effects on humans and animals. This has paved the way for a novel field, ‘nanotoxicology’, that studies the harmful and adverse effects of nanoparticles. Due to the growing availability of nanomaterials, the need for research in nanotoxicology is increasing as human exposure to nanoparticles increases. Despite the advantages, the toxicology of AgNPs is an important consideration in their application. However, as animal testing is costly, time-consuming, and raises ethical concerns, the brine shrimp lethality assay using *Artemia salina* (*A. salina*) larvae is a much simpler, faster, and cheaper alternative for toxicity screening.^30^

The main purpose of this study was to develop and evaluate a sustainable, plant-based approach to synthesise biologically active AgNPs using leaf extracts from five varieties of *T. fournieri*, and to assess their potential in environmental and biomedical applications. To achieve this goal, the Total Phenolic Content (TPC), Total Flavonoid Content (TFC), and Total Antioxidant Content (TAC) were investigated to understand their antioxidant activity and their role in AgNP synthesis. The catalytic activity of *T. fournieri* AgNPs was determined by their ability to reduce PNP, a common pollutant from industries, and the cytotoxicity was assessed against *A. salina* using the brine shrimp lethality assay (BSLA) to ensure that the synthesised AgNPs were non-toxic. The antibacterial activity was evaluated against *Staphylococcus aureus* (*S. aureus*) and *Escherichia coli* (*E. coli*). In addition, the interactions of a single Ag atom and a 3-atom Ag cluster with a target protein were analysed using molecular docking (AutoDock Tools 4.2). The morphology of the synthesised AgNPs was characterised by scanning electron microscopy (SEM). The results provide insights into the applicability of *Torenia*-derived AgNPs in environmental remediation and biomedical fields, support the development of sustainable, plant-based methods for nanoparticle synthesis, and contribute to advancing AgNPs as potential therapeutic agents.

## 2 Experimental

### 2.1 Materials

4-Nitrophenol (PNP) (CAS-100-02-7), aluminium chloride (CAS-7446-70-0), ammonium molybdate (CAS-12027-67-7), chloroform (CAS-67-66-3), ethanol (CAS-64-17-5), ferric chloride (CAS-7705-08-0), filtered seawater, Folin–Ciocalteu (CAS-109001-34-3), gentamycin disc (CAS-1403-66-3), glacial acetic acid (CAS-64-19-7), hydrochloric acid (CAS-7647-01-0), iodine (CAS-7553-56-2), Millon's reagent (CAS-10045-94-0), Molisch's reagent (CAS-90-15-3), Mueller Hinton agar (CAS-9002-18-0), potassium acetate (CAS-127-08-2), saline solution (CAS-7647-14-5), silver nitrate (CAS-7761-88-8), sodium borohydride (CAS-16940-66-2), sodium hydroxide (CAS-1310-73-2), sodium nitrite (CAS-632-00-0), sodium phosphate (CAS-7664-93-9), sulphuric acid (CAS-7664-93-9).

### 2.2 Instruments

Aerator, Analytical Balance (OHAUS®), Autoclave (BIOBASE), Biosafety Cabinet (Heal Force®), Bunsen Burner, Centrifuge (Grant-bio) (80-2B), Fume Hood (BIOBASE), Hot-air Oven (Meditry), HUAWEI Matebook D 16 (12th Gen Intel(R) Core(TM) i5-12450H (2.00 GHz)), Incubator (Thermo SCIENTIFIC BB15 CO_2_ incubator), Light Microscope (LABOMED®), Micropipette (NICHIRYO Nichipet EXII), Refrigerator (Haier), SEM (Hitachi SU6600), Spectrophotometer (JENWAY 6305).

### 2.3 Preparation of water extracts

Five varieties of *T. fournieri* (Fig. S1) were obtained from Diyatha Uyana, Battaramulla, Sri Lanka. The leaves were first shade-dried for 2 days and then oven-dried at 60 °C for 24 hours.

The dried leaves were cut into smaller pieces, and 2 g were added to different beakers, all containing 50 mL of distilled water (DW). The beakers were covered and placed in a dry oven for phytochemical extraction at 65 °C for 60 minutes. The samples were cooled to room temperature (RT), then filtered through Whatman No. 1 filter papers to obtain water extracts (WEs). The absorbance was measured from 320 to 520 nm using DW as the blank. The WEs were stored at 4 °C for further experiments.^31^

### 2.4 Preparation of Ag-nanoparticles

1 mL of WEs was added to 9 mL of 8 mM silver nitrate. Synthesis was carried out at 60 °C and 90 °C for 15, 30, 45, and 60 minutes, and at RT for 24 hours. The absorbance was measured from 320 to 520 nm using DW as the blank. The AgNPs were stored at 4 °C for further experiments.^32^

### 2.5 Methods for analysis of studied compounds

#### 2.5.1 Physicochemical methods

##### 2.5.1.1 Scanning electron microscopy analysis

2 mL of the Lemondrop_AgNPs sample was centrifuged at 10 000 rpm for 2 minutes. The supernatant was discarded, and the pellet was dried at 40 °C for 24 hours. The sample was gold-coated with a sputter coater before analysis. The sample was analysed at the Sri Lanka Institute of Nanotechnology (SLINTEC).

##### 2.5.1.2 Analysis of total flavonoid content (TFC)

2 mL of the WE/AgNP (diluted 15 times) was added to 0.1 mL 10% aluminium chloride and 0.1 mL of 0.1 mM potassium acetate in triplicate. It was incubated at RT for 30 minutes, and the absorbance was measured at 415 nm using DW as the blank. The TFC was calculated using the Quercetin standard curve and expressed as µg/quercetin/100 g.^33^

##### 2.5.1.3 Analysis of Total Phenolic Content (TPC)

1.6 mL of the WE/AgNP (diluted 15 times) was added with 0.4 mL of 10% Folin–Ciocalteu reagent and shaken thoroughly for 3 minutes. 0.2 mL of sodium phosphate was added and incubated at 90 °C for 90 minutes. The absorbance was measured at 760 nm using DW as the blank, and TPC was calculated using the gallic acid standard curve and expressed as (g/gallic acid/100 g).^33^

##### 2.5.1.4 Analysis of Total Antioxidant Content (TAC)

0.5 mL of WE/AgNP (diluted 15 times) was added to 1.5 mL of phospho-molybdenum reagent containing 0.6 M sulphuric acid, 28 mM sodium phosphate, 4 mM ammonium molybdate, mixed in equal parts. This was performed in triplicate. Afterwards, it was incubated at 95 °C for 90 minutes. The absorbance was taken at 695 nm using DW as the blank, and TAC was calculated using the ascorbic acid standard curve expressed as (g/ascorbic acid/100 g).^34^

### 2.6 Catalytic studies

The absorbance of 0.1 mM PNP was measured from 280 to 540 nm using DW as the blank. To 2 mL of 0.1 mM PNP, 1 mL of 0.1 M sodium borohydride was added, and the absorbance was measured for three 10 minutes intervals. To assess PNP catalysis by AgNPs, 5 µL, 50 µL, 60 µL, and 70 µL of 4000 ppm AgNPs were added to 2 mL of 0.1 mM PNP and 1 mL of 0.1 M sodium borohydride, and the absorbance was taken till PNP degradation was noted.^35^

### 2.7 Analysis of biological activity

#### 2.7.1 Antibacterial susceptibility test

The antibacterial activity of AgNPs and WEs was determined using the well diffusion method using bacterial strains of *S. aureus* and *E. coli*. The UV-sterilised Mueller–Hinton Petri dishes were swabbed with the bacterial strains, and three wells were created on the agar for the negative control and two sample duplicates (S1 and S2). A 0.9% saline solution was used as the negative control, and a 30 µg gentamycin antibiotic disc was used as the positive control. AgNPs and WEs were added to S1 and S2 wells, and the plates were incubated at 37 °C for 24 hours. The zones of inhibition (ZOI) were measured.

#### 2.7.2 Determination of cytotoxicity using brine shrimp lethality assay (BSLA)

A spoonful of *A*. *salina* oocytes was allowed to hatch in filtered seawater for 24–36 hours at RT in a well-lit, aerated beaker. The assay was performed in a 96-well plate, and cytotoxicity was determined for 200 ppm and 800 ppm of AgNPs. Each well contained 2 shrimp, and the test was conducted in triplicate with seawater and 2 shrimp as the positive control. The viability was checked 24 hours later using eqn (1).^30^1
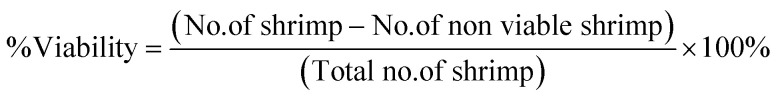


#### 2.7.3 *In silico* molecular docking studies

The 3T1I protein was obtained from the Protein Data Bank (PDB), and the Ag atom, and a 3-atom cluster were modelled and optimised using Avogadro software 1.2.0 and then prepared for docking using AutoDock Tools 4.2. The grid box was centred around the amino acid residues of the NBS1-protein binding site at *X* = 96.583, *Y* = 67.852, and *Z* = 49.048 with 0.744 Å spacing, and the grid dimensions were set to 80 × 100 × 70 along the *X*, *Y*, and *Z* axes. The AutoDock results were analysed using Biovia Discovery Studio 2024 Client.^25^

## 3 Results and discussion

Conventional methods of AgNP synthesis face several issues due to the use of high temperatures and hazardous chemicals, which lead to toxic by-products. Thus, contributing to pollution and health hazards. However, the use of greener alternatives, such as plant extracts, for AgNP synthesis is more advantageous than chemical, physical, and other biological approaches that use microorganisms, due to their minimal environmental impact and improved sustainability.^36^ Water is a safe, inexpensive, and eco-friendly solvent for extracting plant metabolites, emphasising its potential as a sustainable alternative for nanoparticle synthesis. This finding is particularly significant in the context of green nanotechnology, as it aligns with the principles of reducing hazardous chemicals while maintaining efficiency.^37^

### 3.1 Preparation of Ag-nanoparticles

**Fig. 1:**
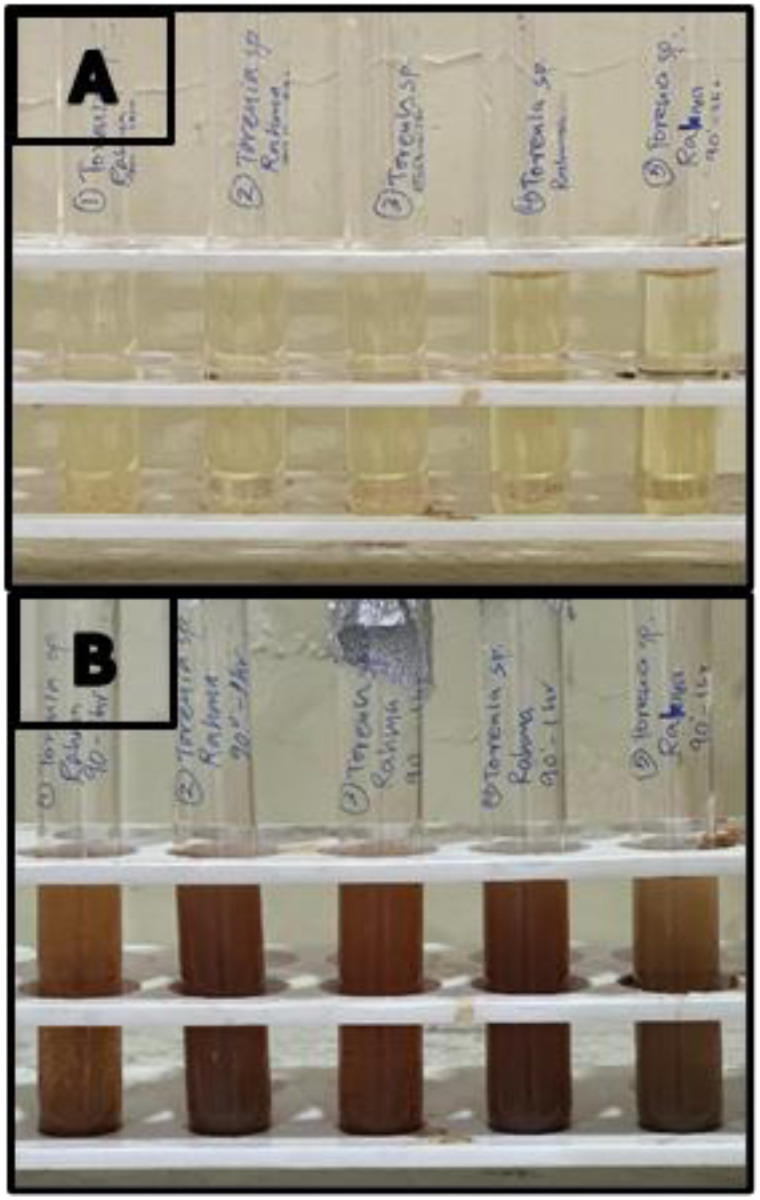
Colour change of the sample heated to 90 °C for 60 minutes from pale yellow to reddish-brown. (A) – Before synthesis, (B) – after synthesis.

Phytochemicals serve as secondary metabolites that act as reducing agents, facilitating the reduction of Ag^+^ to Ag^0^ and as capping agents, preventing Ag atoms from agglomerating, thereby ensuring the stability and formation of colloidal AgNPs.^38,39^ The formation of AgNPs during the synthesis process was confirmed by a colour change from pale yellow to reddish-brown (Fig. 1). This colour change is due to a phenomenon—surface plasma resonance (SPR), which occurs when free electrons between the conduction and valence bands lying close to each other on the surface of AgNPs oscillate in sync with light.^40^

This study demonstrates, for the first time, that water extraction of *T. fournieri* is capable of yielding bioactive phytochemicals with the presence of successful AgNP formation, similar to conventional solvents such as petroleum ether, toluene, chloroform, acetone, and hydroalcoholic extracts previously reported for *Torenia crustacea*, a plant belonging to the same genus, by a previous study.^41^

**Fig. 2:**
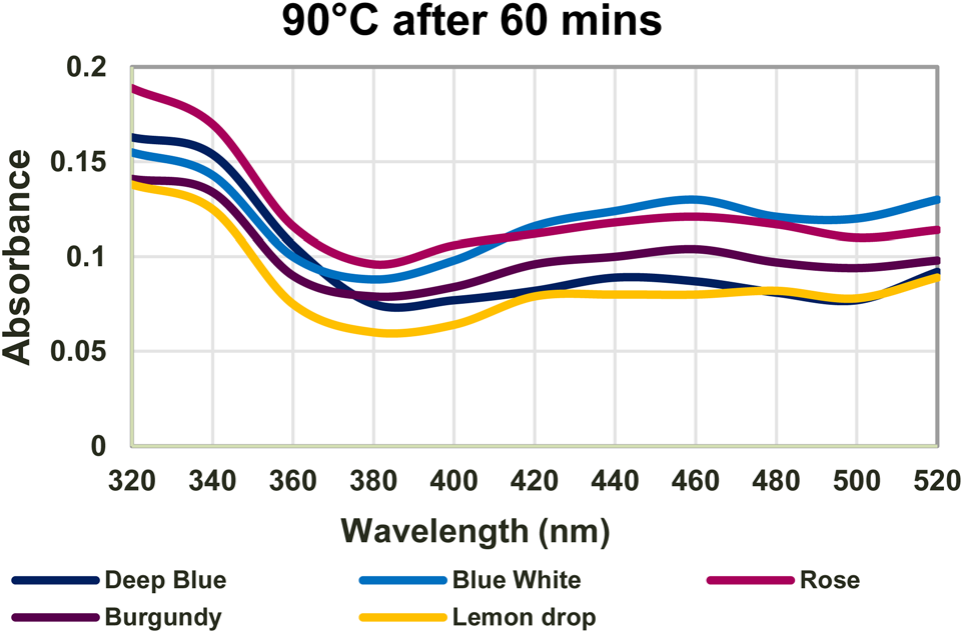
Absorbance graph of optimised AgNPs with AgNP peaks.

AgNP synthesis can be influenced by pH, silver nitrate concentration, temperature, and time. In this study, synthesis was performed at temperatures of 60 °C and 90 °C for 15, 30, 45, and 60 minutes, as well as at RT for 24 hours, and AgNPs were synthesised at all tested temperatures and times. Among these, the reaction carried out at 90 °C for 60 minutes produced the most intense reddish-brown colour (Fig. 1), and prominent SPR peaks visible in the UV-visible spectrum (Fig. 2). Hence, the optimised conditions for AgNP synthesis in this study were incubating at 90 °C for 60 minutes.

#### 3.1.1 Ag-nanoparticle characterisation

In UV characterisation of AgNPs, absorption peaks corresponding to this SPR phenomenon can be detected between 400 and 500 nm.^42^ Synthesised AgNPs showed peaks at 440 nm for Deepblue_AgNPs, 460 nm for Bluewhite_AgNPs, Burgundy_AgNPs, and Rose_AgNPs, and 480 nm for Lemondrop_AgNPs, indicating the presence of AgNPs. However, UV spectral data for WEs did not show any peaks from 320–520 nm.

**Fig. 3:**
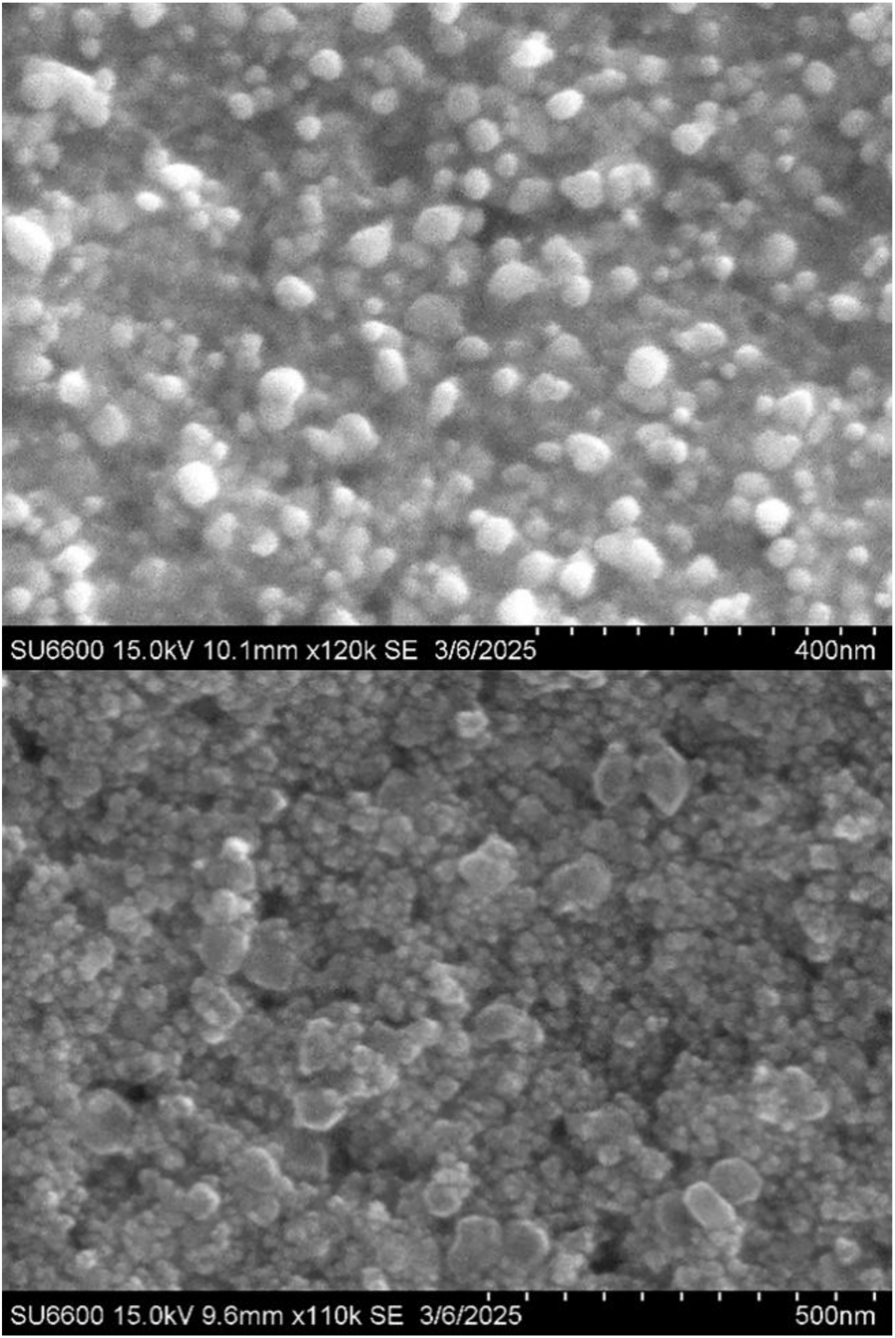
SEM images of Lemondrop_AgNPs 15.0 kV 10.1 mm ×120k SE at 400 nm (top) and 15.0 kV 9.6 mm ×110k SE at 500 nm (bottom). SEM images reveal spherical 25–60 nm Lemondrop_AgNPs.

SEM characterisation was done to analyse the composition and morphology of the synthesised AgNPs.^43^ SEM results revealed a predominant spherical morphology of 25 to 60 nm Lemondrop_AgNPs (Fig. 3). SEM analysis of a previously conducted nanoparticle study on the whole plant ethanolic extracts of *T*. *crustacea* also reported spherical-shaped, non-uniform distribution of AgNPs with a mean size of about 16 nm.^44^ Nanoparticles of this size are known to exhibit a high surface-area-to-volume ratio, with enhanced catalytic and antibacterial properties, utilised in drug delivery. Further, their small size allows for easy penetration into cell membranes.^45^

This is due to higher antioxidant content which leads to better capping and stabilization of nanoparticles that results in smaller nanoparticles, whereas lower antioxidant concentrations, causes more aggregation of formed nanoparticles, leading to larger particle sizes.^17^

Band gap energy calculation is an important parameter in assessing the conductivity of AgNPs. It is the energy difference required for an electron to move from the conduction band to the valence band and can be calculated using Planck's equation. Materials can be classified as insulators or semiconductors when band gaps are greater than 4 eV and less than 3 eV, respectively.^46^ All synthesised AgNPs are semiconductors as calculated band gaps are less than 3 eV (Table 1), and can be used for photocatalysis, photo-optics, and electronics.^47^

**Table 1:** Data representation for antioxidant analysis, bandgap energy and PNP consumption rates

	Deep blue	Blue white	Rose	Burgundy	Lemondrop
TFC (µg/QE/100 g)	676.04 ± 2.604^a^	666.93 ± 7.92^a^	1182.55 ± 41.91^a^	970.31 ± 0^a^	1413.02 ± 74.127^a^
	10901.042 ± 26.04^b^	12281.25 ± 157.869^b^	11734.38 ± 238.675^b^	14026.04 ± 101.696^b^	13856.77 ± 56.756^b^
TPC (g/GAE/100 g)	2173.71 ± 27.041^a^	2135.86 ± 31.556^a^	2245.86 ± 15.105^a^	2131.57 ± 7.734^a^	2169.43 ± 6.888^a^
	6980 ± 21.724^b^	7651.43 ± 117.097^b^	7694.29 ± 110.02^b^	7780 ± 182.387^b^	8487.14 ± 191.79^b^
TAC (g/GAE/100 g)	222.76 ± 5.355^a^	198.78 ± 3.252^a^	235.60 ± 1.537^a^	236.62 ± 3.645^a^	269.69 ± 8.872^a^
	456.02 ± 7.954^b^	458.30 ± 12.806^b^	481.02 ± 13.777^b^	525.34 ± 7.872^b^	565.11 ± 7.954^b^
Bandgap energy (eV)	2.82	2.70	2.70	2.70	2.58
PNP consumption rate (*k*) (min^−1^)	0.0211	0.0146	0.0118	0.0162	0.0165

a Water extract

b AgNPs

These extract-mediated effects on particle size directly impact the optical behaviour of AgNPs. The band gap energy of AgNPs is known to be strongly size-dependent, with several studies reporting an inverse relationship between nanoparticle size and band gap energy. These findings demonstrate that the chemical constituents of the WEs indirectly modulate the band gap energy of AgNPs by controlling nanoparticle size and growth behaviour. The observed semiconducting characteristics with a relatively large band gap highlight the potential applicability of the biosynthesised AgNPs in optoelectronic devices, sensing applications, and energy storage systems.^48^

#### 3.1.2 Antioxidant assays

Phenolic compounds such as polyphenols and flavonoids found in medicinal plants have antioxidant properties.^49^ TFC, TPC, and TAC antioxidant assays were performed to assess the antioxidant content present in AgNPs and WEs (Fig. S2). To date, no research has evaluated the antioxidant activity of *Torenia* sp.-based AgNPs.

The aluminium chloride colourimetric assay was done to determine TFC in AgNPs and WEs. The principle of this assay relies on the formation of an acid–stable complex between Al^3+^ and the C-4 keto group of the flavonoid and either its C-3 or C-5 hydroxyl group. Due to the presence of many oxo and hydroxyl groups, flavonoids bind with Al^3+^ with great affinity. The absorbance maximum of this chelate complex is around 400 nm in UV-visible spectroscopy.^50^ AgNPs showed higher TFC than WEs in the order Burgundy_AgNPs > Lemondrop_AgNPs > Bluewhite_AgNPs > Rose_AgNPs > Deepblue_AgNPs (Table 1). Calculated *F*-value > *F*-crit and *p*-value < 0.05, concluding a significant difference for TFC values in AgNPs and WEs.

To quantify TPC, the Folin–Ciocalteu assay was performed. The Folin–Ciocalteu reagent contains tungsten and molybdenum. Electron transfer from phenolic compounds to phosphomolybdic or phosphotungstic acid complexes under alkaline conditions gives a colour change from yellow to blue, and was observed during the study. This occurs due to the reduction of the reagent, which can be spectrophotometrically measured at 760 nm.^51^ TPC was higher in AgNPs than in WEs in the order Lemondrop_AgNPs > Burgundy_AgNPs > Rose_AgNPs > Bluewhite_AgNPs > Deepblue_AgNPs (Table 1). The calculated *F*-value > *F*-crit and *p*-value < 0.05 conclude a significant difference in TPC values for AgNPs and WEs.

The Phosphomolybdenum method was used to determine TAC. This follows either an electron transfer or hydrogen atom transfer mechanism, reducing molybdenum(vi) to molybdenum(v) by the antioxidants present in the sample. This results in a greenish–blue complex with maximum absorbance at 695 nm.^52^ AgNPs had higher TAC than in WEs in the order Lemondrop_AgNPs > Burgundy_AgNPs > Rose_AgNPs > Bluewhite_AgNPs = Deepblue_AgNPs (Table 1). Calculated *F*-value > *F*-crit and *p*-value < 0.05, concludes a significant difference in TAC values for AgNPs and WEs.

Pearson correlation was done to assess the relation among the antioxidants, and a very strong positive correlation was observed between TFC–TAC (0.985), TFC–TPC (0.996) and TPC–TAC (*r* = 0.989). This also suggests that phenolic content contributed to a stronger overall antioxidant activity, which is in line with previously conducted research on the flowers of *Torenia* sp. according to a previous study.^4^

In a previous study, FTIR analysis on AgNPs of the ethanolic extract of *T. crustacea* confirmed the presence of alkenes, aromatics, alkyl halides, esters, ketones, amides, and aromatic functional groups.^44^ The availability and concentration of these bioactive compounds determine the final particle size. Higher amounts of reducing agents lead to the formation of smaller AgNPs. Simultaneously, capping molecules adsorb onto the surface of the nanoparticle, restricting further growth and minimising aggregation. By contrast, insufficient stabilisation results in larger particle sizes.^17^

### 3.2 Catalytic properties of Ag-nanoparticles

The absorbance maximum of PNP is between 300 and 320 nm. The reduction of PNP/4-NP to PAP/4-AP using sodium borohydride, a mild reducing agent, occurs in a two-step reaction and is visible due to a shift in absorbance maximum. First, sodium borohydride donates hydride ions (H^−^) to the nitro group of PNP, converting it to *p*-nitrophenolate (4-NP^−^). This is visible due to a peak shift from 320 nm to 400 nm (Fig. S3) and a colour change from a light yellow solution to a bright yellow solution.^19^ Next, hydrogenation of 4-NP^−^ forms a colourless 4-AP solution,^53^ forming a new PAP peak around 300 nm.^54^

The second reaction is slow, requiring high temperatures or longer reaction times to achieve complete conversion. In the presence of biocatalysts such as AgNPs, the adsorption of both PNP and sodium borohydride is facilitated onto the nanoparticle surface, thereby accelerating electron transfer by increasing stability, lowering the activation energy, and leading to a faster and more efficient conversion due to their unique electronic properties and high surface area.^35^

**Fig. 4:**
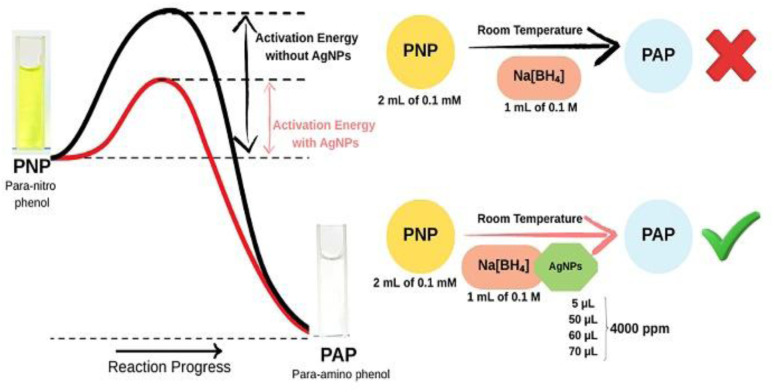
Experimental scheme for PNP catalysis study.

In this study (Fig. 4), PNP did not degrade in the presence of NaBH_4_ alone. Slight degradation of PNP was noted when 70 µL of Deepblue_AgNPs was added. The experiment was repeated separately with 60 µL and 50 µL of Deepblue_AgNPs, and similarly, partial degradation was noted (Fig. S4). Therefore, the experiment was repeated with a much smaller volume, such as 5 µL of AgNPs, and PNP was completely degraded within 45 minutes (Fig. S5).

A high concentration of AgNO_3_ (8 mM) was used to synthesise AgNPs in this study, which may have resulted in the agglomeration of nanoparticles when a higher volume of AgNPs was added.^55^ However, as reported by Bélteky *et al.* there is a research gap in understanding the agglomeration of AgNPs.^56^

PNP catalysis follows pseudo-first-order kinetics and the rates were calculated using (eqn (2)),2
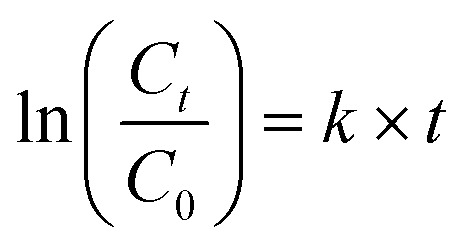
where *C*_*t*_ = Peak concentration, and *C*_0_ = Initial concentration, *k* = rate constant, *t* = time.^35^

**Fig. 5:**
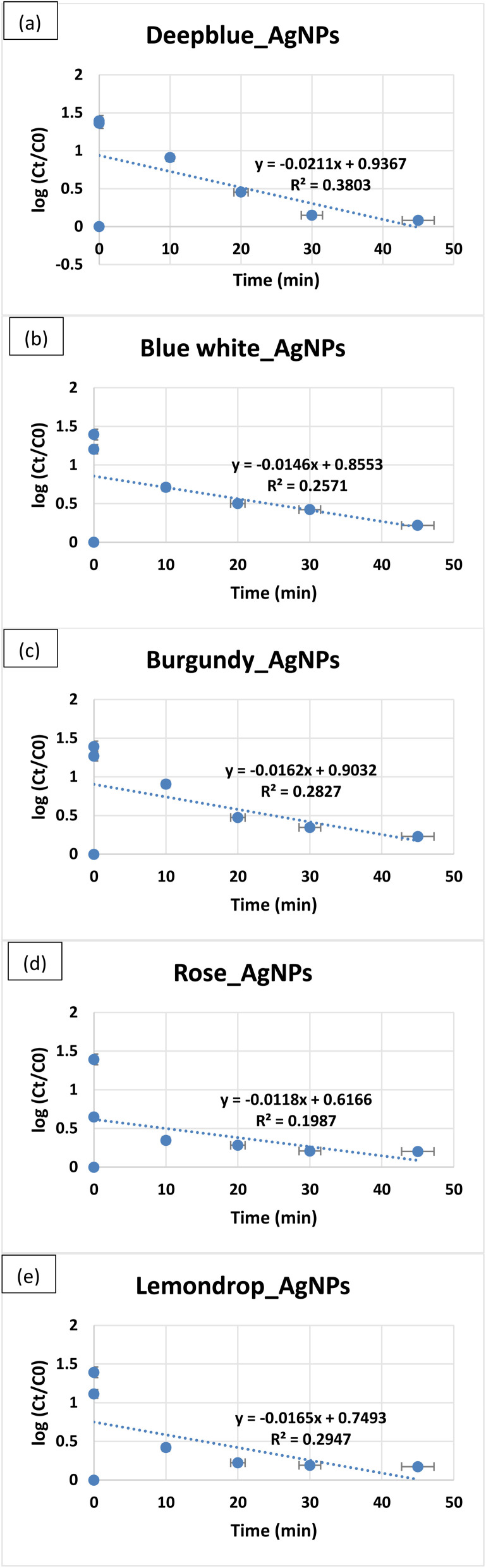
Kinetic curves of PNP consumption; (A)-Deepblue_AgNPs, (B)-Blue white_AgNPs, (C)-Rose_AgNPs, (D)-Burgundy_AgNPs, and (E)-Lemondrop_AgNPs.

The AgNPs degraded PNP in the order Deepblue_AgNPs > Lemondrop_AgNPs > Burgundy_AgNPs > Bluewhite_AgNPs > Rose_AgNPs (Fig. 5) with respect to their *k* values (Table 1).

In the future, PNP catalysis can be performed using different AgNP concentrations to optimise the reaction, and its application can be further tested on biological water bodies polluted with PNP to assess the effectiveness.

### 3.3 Biological activity of Ag-nanoparticles

#### 3.3.1 Antibacterial susceptibility testing

The exact mechanism of the antimicrobial mode of action of AgNPs is unknown. However, AgNPs are known to damage the cell wall and membrane, disrupt their integrity, penetrate cells due to their small size, damage DNA and proteins, and generate ROS, leading to oxidative stress. AgNPs of size in the range of 20 to 80 nm are known to be toxic to microorganisms as they release Ag^+^ ions.^40,57^

In this study, all synthesised AgNPs showed effective antimicrobial activity compared to WEs against Gram-negative (*E. coli*) and Gram-positive (*S. aureus*) bacteria (Fig. S6–S10) as previously documented by Roy *et al.*^58^

*E. coli* had a higher ZOI compared to *S. aureus*, which is in line with previously conducted studies by Loo *et al.*^59^ This is due to the presence of a thick peptidoglycan layer in *S. aureus*, which hinders the penetration of AgNPs, reducing its efficacy compared to *E. coli*, which possesses a thin peptidoglycan layer with a negatively charged outer membrane, facilitating the adhesion of positively charged AgNPs.^46^

#### 3.3.2 Assessing cytotoxicity using brine shrimp lethality assay

**Fig. 6:**
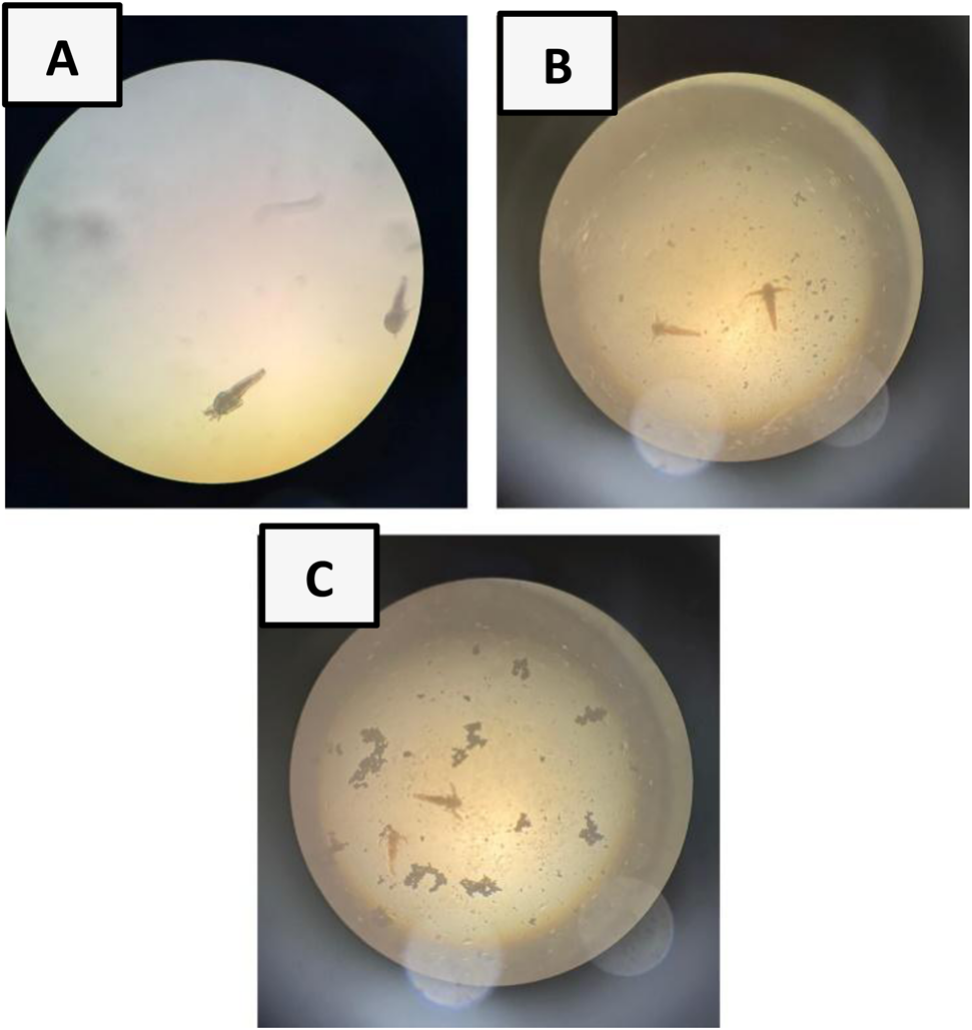
Results of brine shrimp lethality assay (BSLA) 24 hours later: (A)-viable brine shrimp (control), (B)-viable brine shrimp in 200 ppm of Lemondrop_AgNPs, (C)-viable brine shrimp in 800 ppm of Lemondrop_AgNPs (×40).

BSLA is a preliminary screening tool in assessing the cytotoxicity of plant extracts and AgNPs using *A. salina* larvae. It is a model organism for cytotoxic studies due to its cuticle, which is composed of chitin, an important material found in many aquatic organisms, which provides structural support and plays an important role in molting. The cytotoxicity of AgNPs occurs when Ag^+^ released from AgNPs binds with functional groups of chitin, altering the structural integrity and disrupting the molting process.^60^ BSLA results showed 100% viability of brine shrimps at both 800 ppm and 200 ppm AgNPs, showing the non-toxicity of the synthesised AgNPs (Fig. 6).

#### 3.3.3 *In silico* docking studies of an Ag atom and an Ag cluster against MRE11

During docking analysis, a binding energy of less than 0 kcal mol^−1^ is favourable for an Ag atom.^61^ The Ag atom interacted with amino acids PHE13, LEU287, and ILE289 (Fig. S11), while the Ag_2_ cluster interacted with ASP20, GLY58, GLY59, and SER124 (Fig. S12) with a binding energy of −0.19 kcal mol^−1^ and −1.11 kcal mol^−1^, respectively (Tables 2 and 3). Buglak, Ramazanov and Kononov^62^ also reported a lower binding energy for the Ag cluster than for an Ag atom. El-Naggar *et al.* and Akram *et al.* reported similar interactions of Ag with PHE, LEU, ASP, GLY, and SER.^25,61^ However, Ag showed no interactions with any residues of the NBS1-binding site.^27^

**Table 2:** Interactions of Ag atom and their bond lengths, binding energy, and inhibition constant

Amino acids	PHE13	LEU287	ILE289
Bond length (Å)	2.64	2.96	2.52
Binding energy	−0.19 kcal mol^−1^		
Inhibition constant	731.07 mM		

**Table 3:** Interactions of Ag cluster and their bond lengths, binding energy, and inhibition constant

Amino acids	ASP20	GLY58	GLY59	SER124
Bond length (Å)	2.45	2.44	2.44	2.25
Binding energy	−1.11 kcal mol^−1^			
Inhibition constant	154.62 mM			

## 4 Conclusions

In conclusion, 5 varieties of *Torenia fournieri* leaves were used to synthesise AgNPs, and the optimum condition for AgNP synthesis was 90 °C for 60 minutes. TFC, TPC, and TAC antioxidant assays showed higher amounts in AgNPs than in WEs. Deepblue_AgNPs showed the highest rate of degradation (*k* = 0.021 min^−1^) for PNP. AgNPs showed significantly higher ZOI compared to WEs for both *S. aureus* and *E. coli* showing effective antimicrobial activity. 100% viability was recorded in BSLA, showing the non-toxicity of synthesised AgNPs. *In silico* docking studies revealed a lower binding energy with the Ag cluster than the Ag atom, indicating a good binding affinity to the MRE11 protein.

## Author contributions

MK: conceptualization, methodology, funding acquisition, project administration, supervision, validation, writing—review & editing. RA: data curation, formal analysis, investigation, methodology, validation, visualization, writing—original draft. BG: investigation, supervision, validation, visualization, writing—review & editing. OP: supervision, validation, visualization, writing—review & editing. All authors read and approved the submitted version.

## Conflicts of interest

There are no conflicts to declare.

## Supplementary Material

RA-016-D5RA10074G-s001

## Data Availability

The data supporting this article have been included as part of the Supplementary Information (SI). Supplementary information: further experimental details. See DOI: https://doi.org/10.1039/d5ra10074g.
