# Supplementary material for: Green synthesis of silver nanoparticles with Torenia fournieri leaf extracts and assessing the antioxidant and antibacterial properties, para-nitrophenol catalysis, and nanotoxicity
Source: RSC Adv. 2026 Mar 17;16(16):14525–34. doi: 10.1039/d5ra10074g (PMC12993830; doi:10.1039/d5ra10074g)
Supplement: RA-016-D5RA10074G-s001 [file RA-016-D5RA10074G-s001.pdf]

Supplementary Information (SI) for RSC Advances.
This journal is © The Royal Society of Chemistry 2026

Supporting Information

Green Synthesis of Silver Nanoparticles with *Torenia fournieri* leaf extracts and Assessing the Antioxidant, Antibacterial Properties, Para-nitrophenol Catalysis, and Nanotoxicity.

Mathivathani Kandiah[a]*, Rahma Arifeen[a], Beneli Gunaratne[a], Ominda Perera[a]

[a] Faculty of Life and Medical Sciences, Business Management School (BMS) Campus, Colombo 00600, Sri Lanka.

*Corresponding author: mathi@bms.ac.lk

Statistical Analysis -

One-way ANOVA using Microsoft® Excel 2016 software and Pearson correlation was analysed using IBM SPSS Statistics software Version 29.

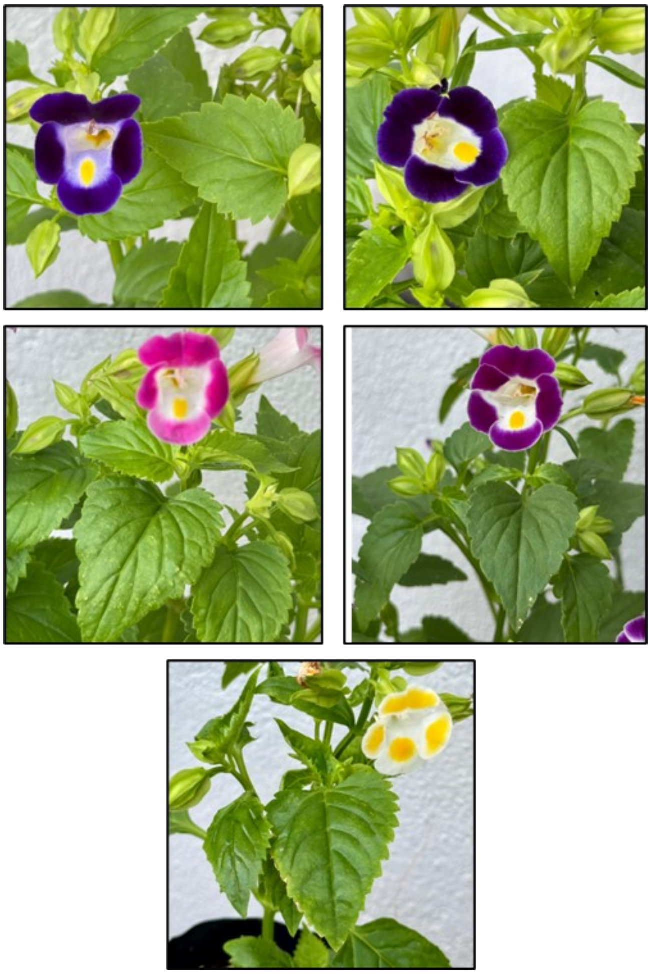

Figure S1. Leaves of five varieties of *Torenia fournieri*: (A) – Deep blue (D.B), (B) – Blue White (B.W), (C) – Rose (R), (D) – Burgundy (B), (E) – Lemondrop (L)

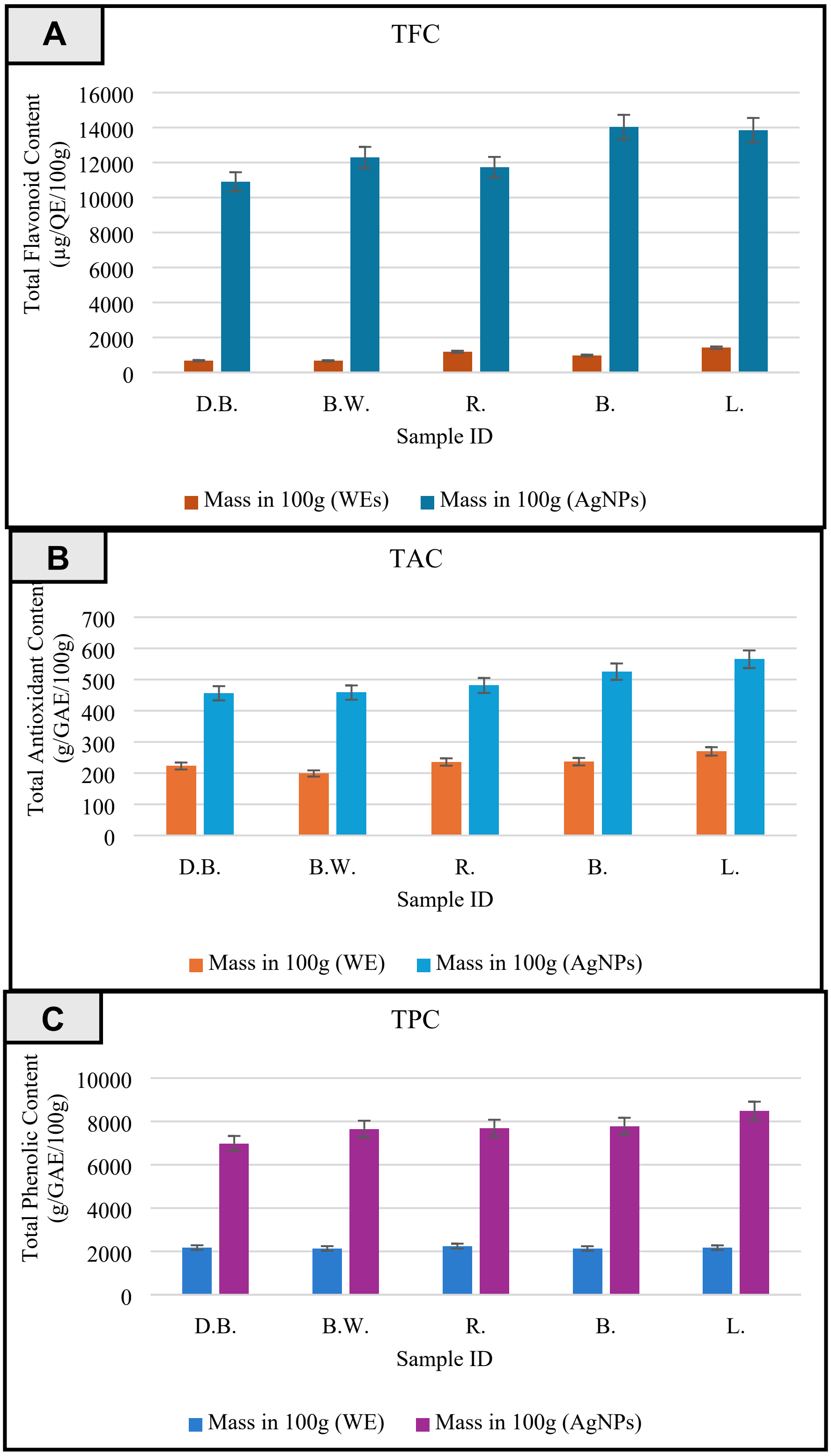

Figure S2. Graphs of tested WEs and AgNPs depicting the amount of (A) – TFC, (B) – TPC, and (C) – TAC.

Table S1 ANOVA test for TFC in WEs and AgNPs

SUMMARY				
Groups	**Count**	**Sum**	**Average**	**Variance**
Mass in 100g (WEs)	5	4908.85	981.77	104754.47
Mass in 100g (AgNPs)	5	62799.48	12559.90	1835598.41

ANOVA						
Source of Variation	**Sum of squares**	**Degrees of freedom**	**Mean square**	**F-statistic**	**P-value (Significance)**	**Critical F-value**
Between Groups	335132446.3	1	335132446.3	345.43	7.24E-08	5.32
Within Groups	7761411.54	8	970176.44			
Total	342893857.8	9				

*Error rate – 95 % confidence with an error rate of 5%

Table S2 ANOVA test for TPC in WEs and AgNPs

SUMMARY				
Groups	**Count**	**Sum**	**Average**	**Variance**
Mass in 100g (WEs)	5	10856.43	2171.29	2100.67
Mass in 100g (AgNPs)	5	38592.86	7718.58	286265.31

ANOVA						
Source of Variation	**Sum of squares**	**Degrees of freedom**	**Mean square**	**F-statistic**	**P-value (Significance)**	**Critical F-value**
Between Groups	76930946.99	1	76930946.99	533.56	1.31E-08	5.32
Within Groups	1153463.88	8	144182.99			
Total	78084410.87	9				

*Error rate – 95 % confidence with an error rate of 5%

Table S3 ANOVA test for TAC in WEs and AgNPs

SUMMARY				
Groups	**Count**	**Sum**	**Average**	**Variance**
Mass in 100g (WEs)	5	1163.47	232.70	660.35

Mass in 100g (AgNPs)	5	2485.80	497.16	2218.75

ANOVA						
Source of Variation	**Sum of squares**	**Degrees of freedom**	**Mean square**	**F-statistic**	**P-value (Significance)**	**Critical F-value**
Between Groups	174855.50	1	174855.54	121.47	4.09E-06	5.32
Within Groups	11516.39	8	1439.55			
Total	186371.9	9				

*Error rate – 95 % confidence with an error rate of 5%

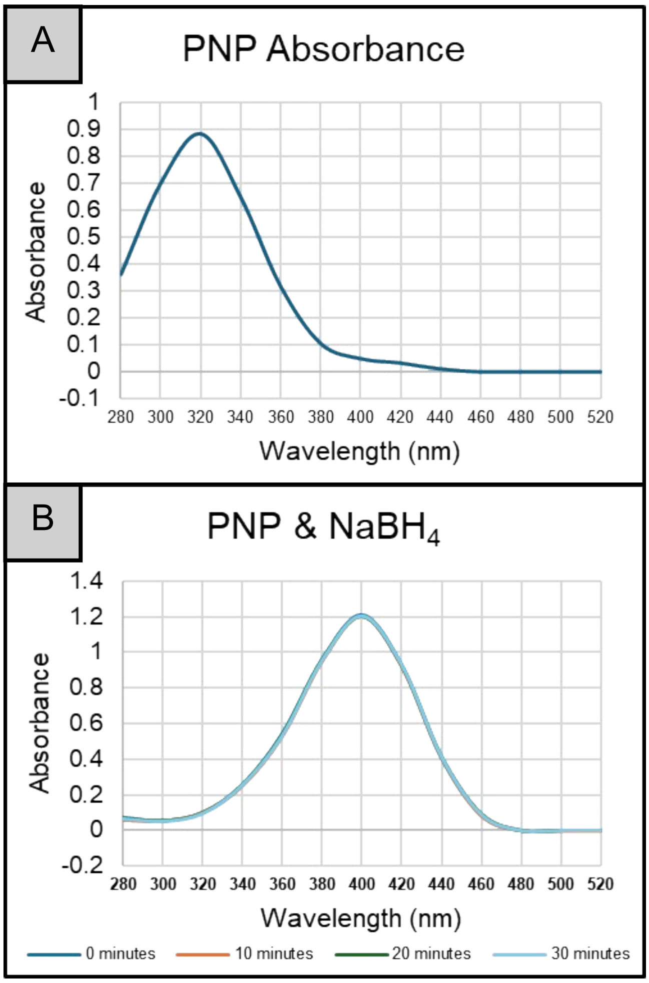

Figure S3. Absorbance curves (A)-PNP (showing maximum absorbance at 320nm) (B)-PNP and $NaBH_4$ (peak shift to 400nm)

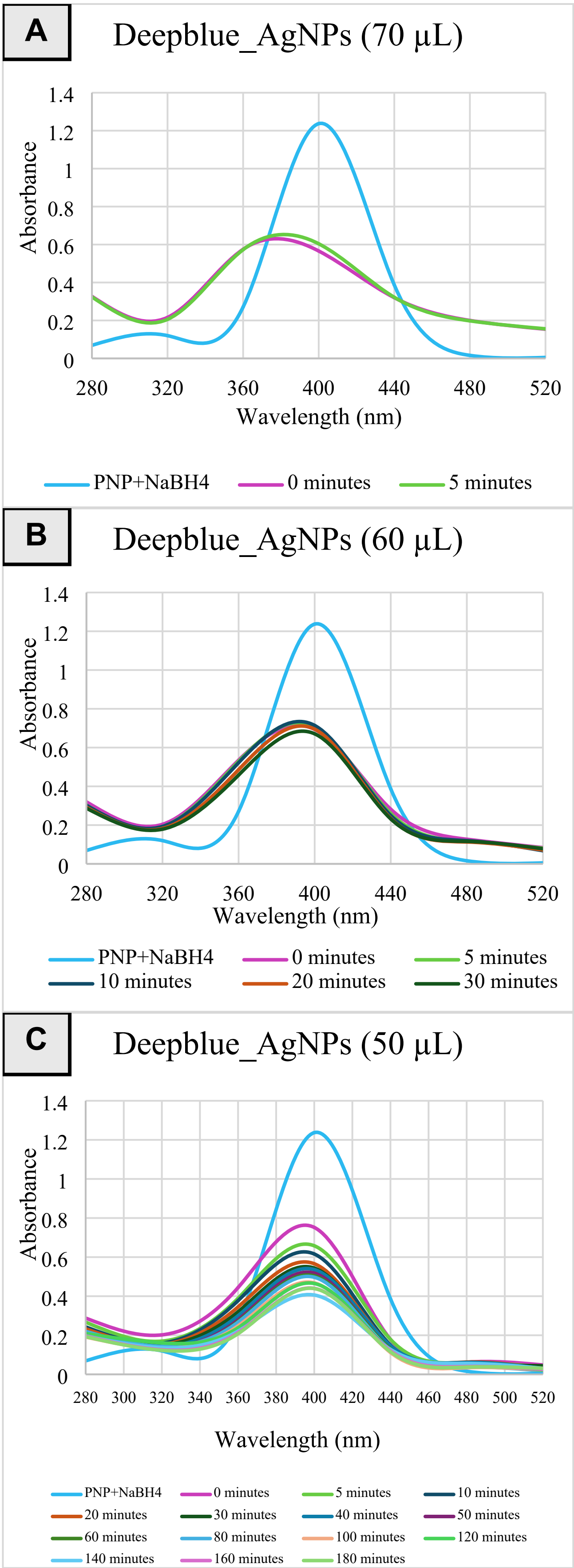

Figure S4. PNP catalysis with 4000ppm Deepblue_AgNPs in the presence of $NaBH_4$, (A) – 70 µL, (B) – 60 µL, and (C) – 50 µL.

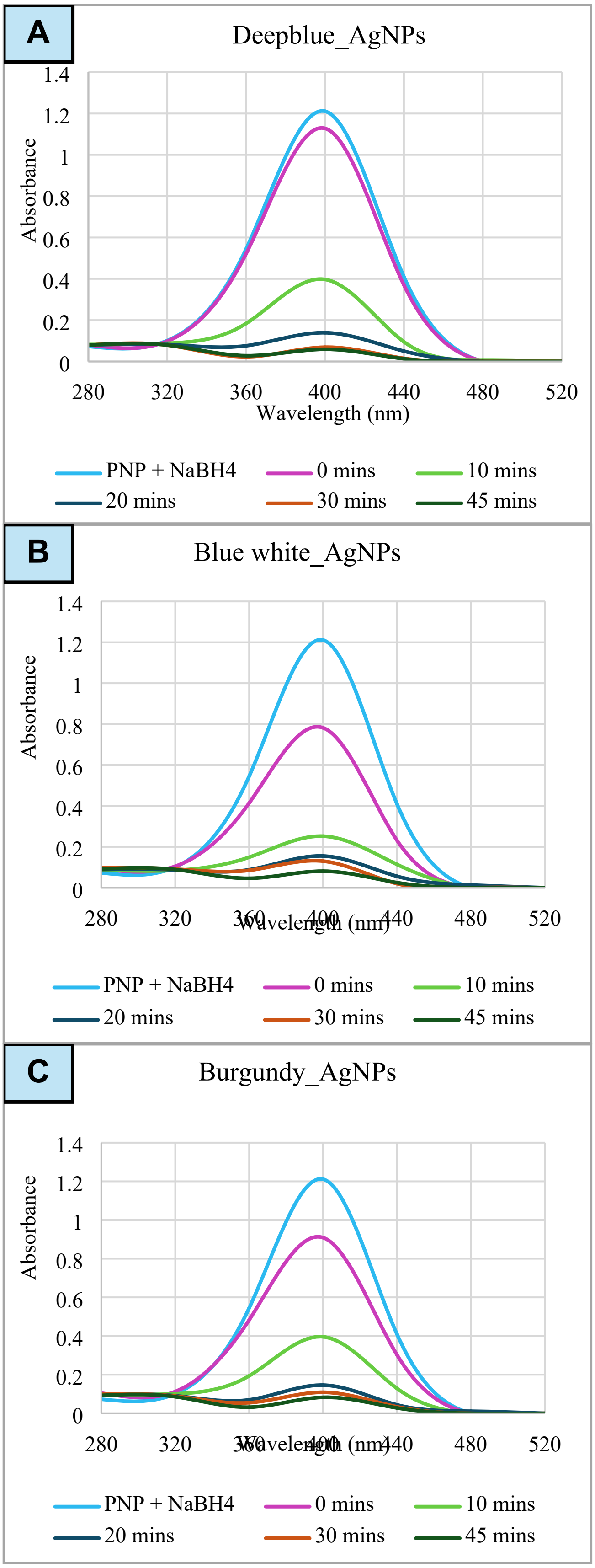
A
Deepblue_AgNPs
Absorbance
1.4
1.2
1
0.8
0.6
0.4
0.2
0
280
320
360
400
440
480
520
Wavelength (nm)
PNP + NaBH4
0 mins
10 mins
20 mins
30 mins
45 mins
B
Blue white_AgNPs
Absorbance
1.4
1.2
1
0.8
0.6
0.4
0.2
0
280
320
360
400
440
480
520
Wavelength (nm)
PNP + NaBH4
0 mins
10 mins
20 mins
30 mins
45 mins
C
Burgundy_AgNPs
Absorbance
1.4
1.2
1
0.8
0.6
0.4
0.2
0
280
320
360
400
440
480
520
Wavelength (nm)
PNP + NaBH4
0 mins
10 mins
20 mins
30 mins
45 mins

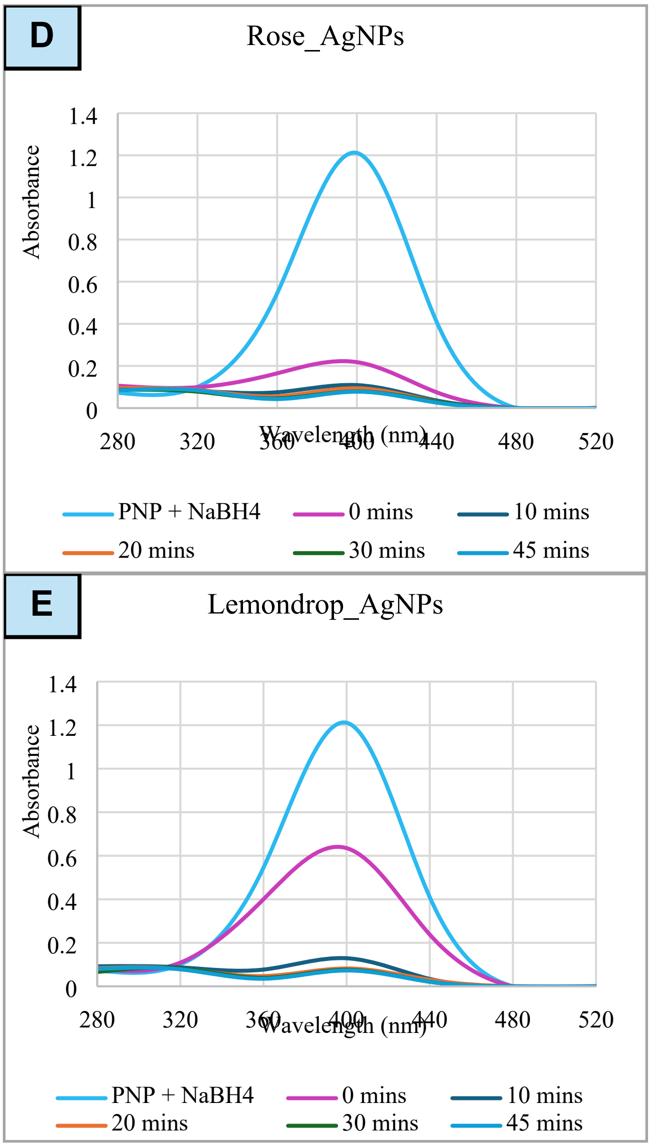

Figure S5. PNP catalysis with 5 μL of 4000ppm AgNPs in the presence of $NaBH_4$. (A)-Deepblue_AgNPs, (B)-Blue white_AgNPs, (C)-Rose_AgNPs, (D)-Burgundy_AgNPs, and (E)-Lemondrop_AgNPs

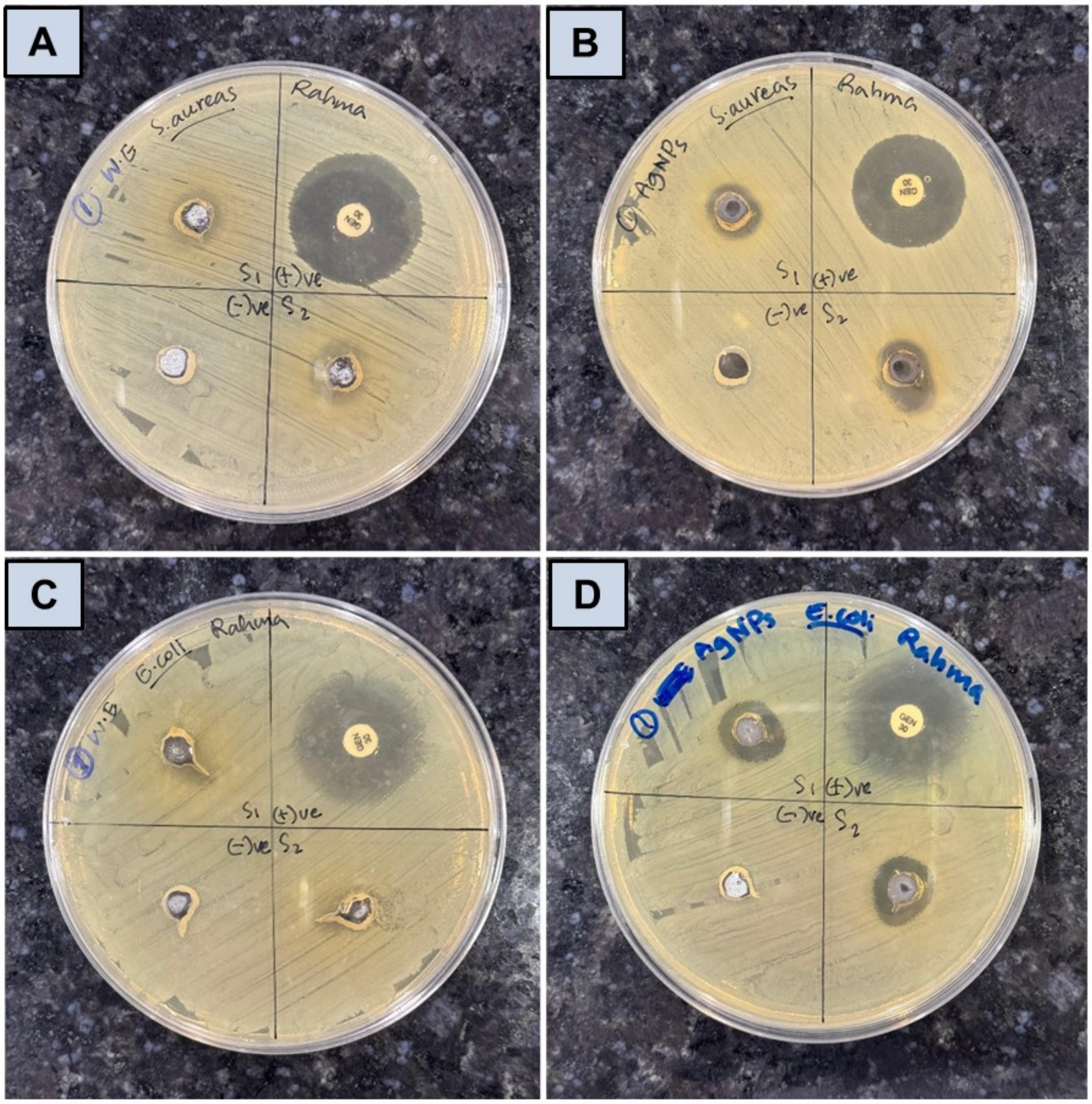

Figure S6. ZOIs of DeepBlue (A)-WE against *S. aureus* (B)-AgNPs against *S. aureus* (C)-WE against *E. coli* and (D)-AgNPs against *E. coli.*

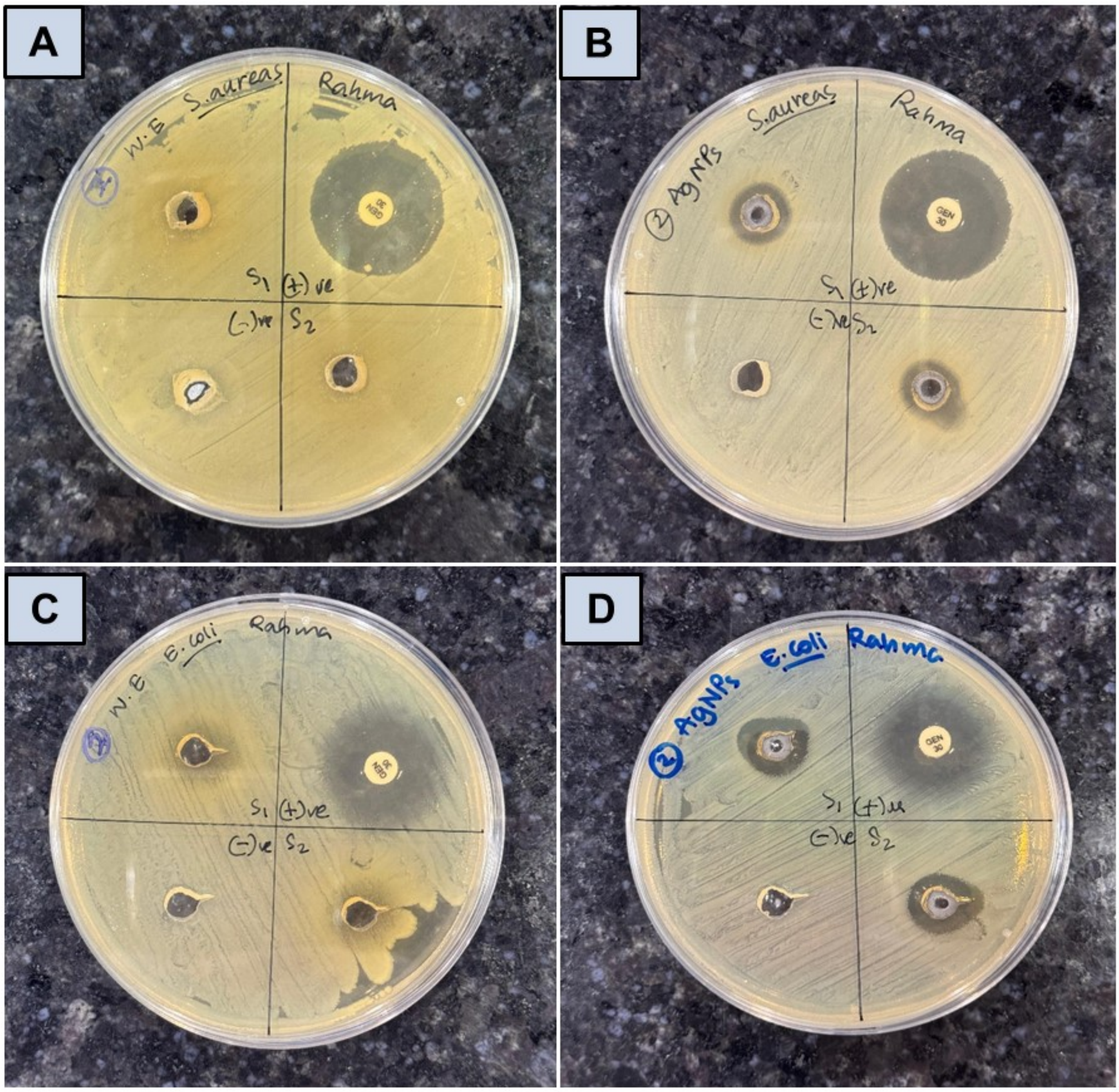

Figure S7. ZOIs of Blue white (A)-WE against *S. aureus* (B)-AgNPs against *S. aureus* (C)-WE against *E. coli* and (D)-AgNPs against *E. coli.*

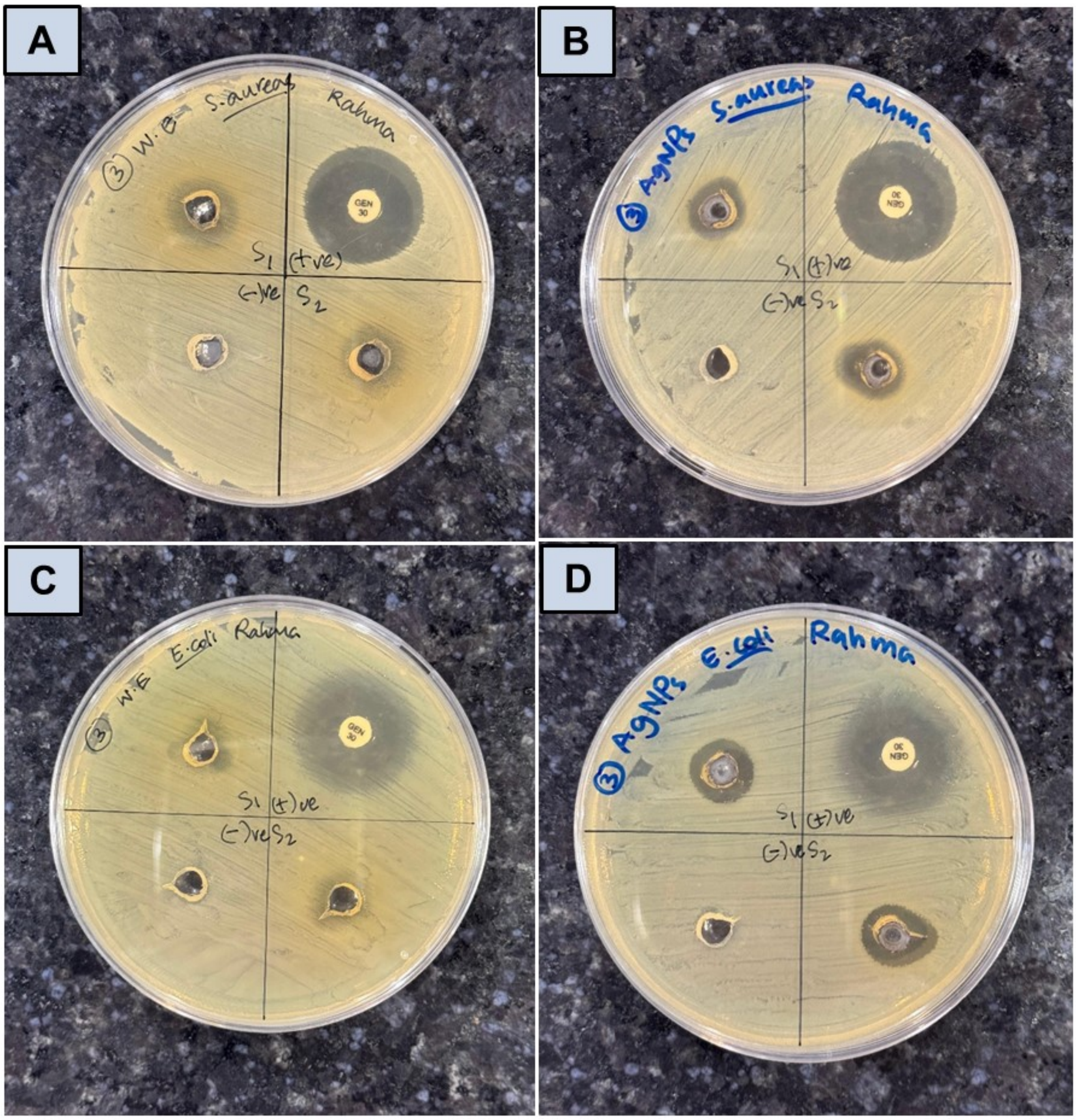

Figure S8. ZOIs of Rose (A)-WE against *S. aureus* (B)-AgNPs against *S .aureus* (C)-WE against *E. coli* and (D)-AgNPs against *E. coli.*

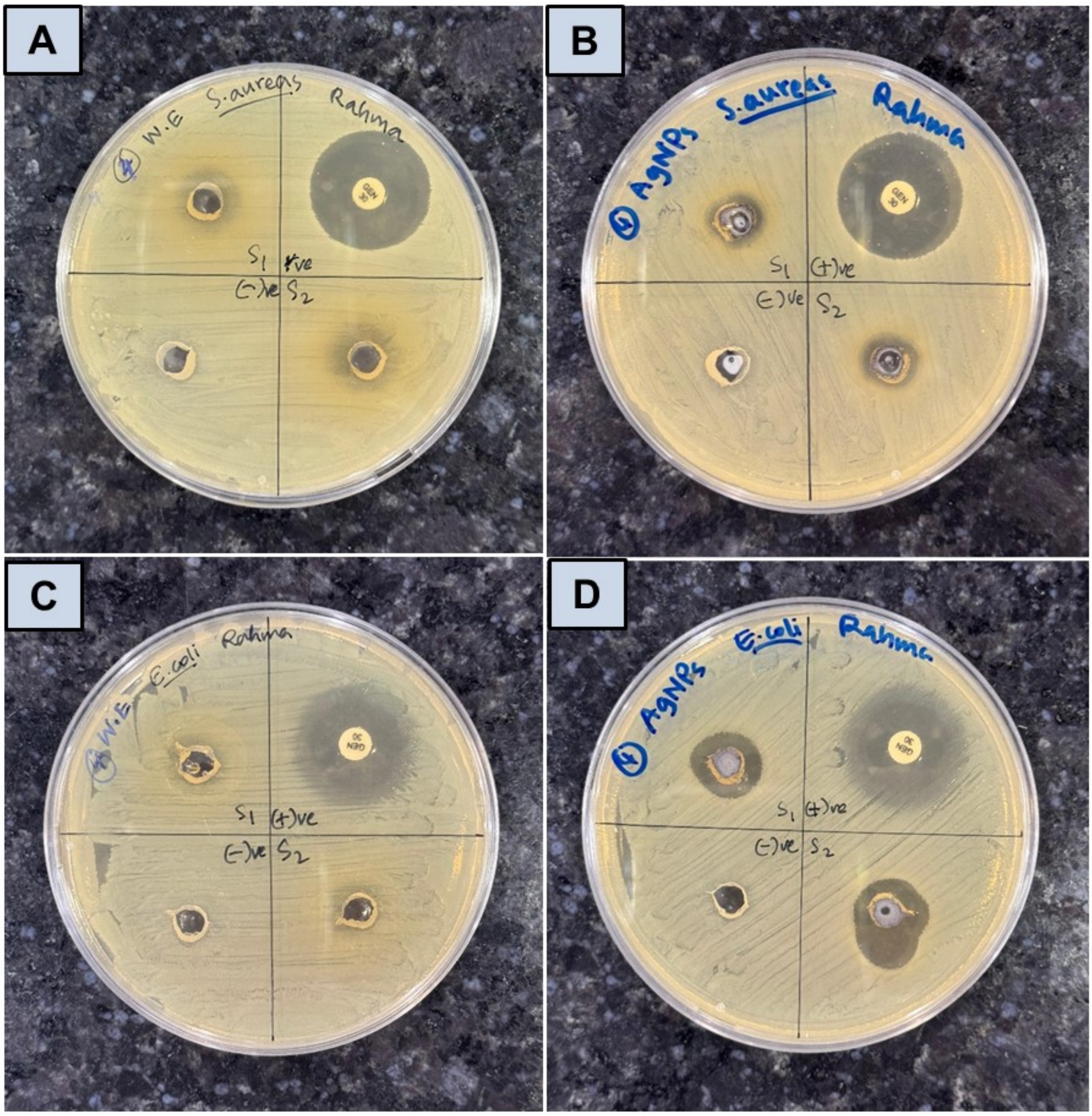

Figure S9. ZOIs of Burgundy (A)-WE against *S. aureus* (B)-AgNPs against *S .aureus* (C)-WE against *E. coli* and (D)-AgNPs against *E. coli.*

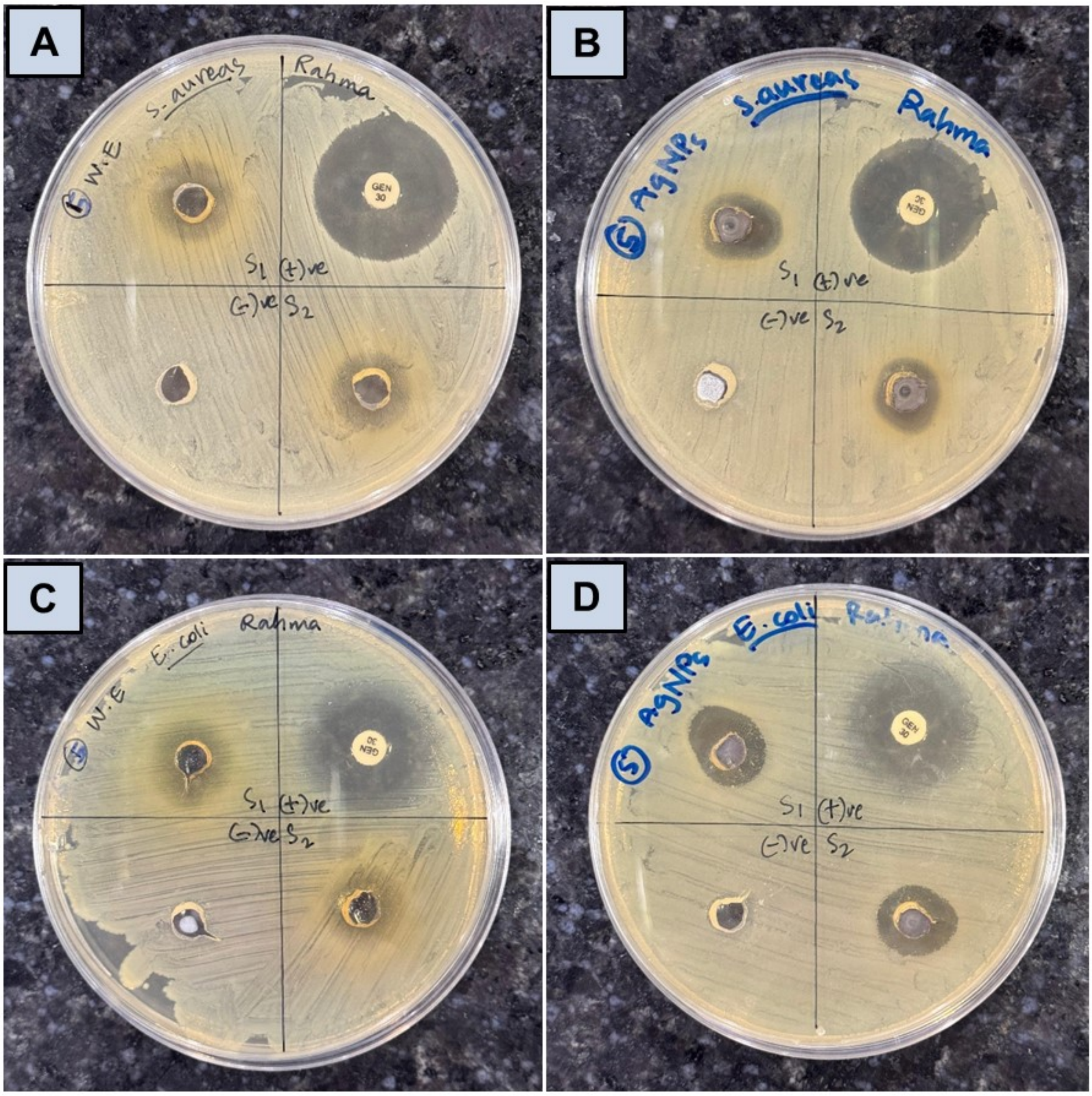

Figure S10. ZOIs of Lemondrop (A)-WE against *S. aureus* (B)-AgNPs against *S .aureus* (C)-WE against *E. coli* and (D)-AgNPs against *E. coli.*

Table S4 ANOVA test of WEs and AgNPs against *S.aureus*

SUMMARY						
Groups	**Count**	**Sum**		**Average**		**Variance**
WEs	5	3.75		0.75		0.005
AgNPs	5	5.7		1.14		0.003
ANOVA						
Source of Variation	**Sum of squares**	**Degrees of freedom**	**Mean square**	**F-statistic**	**P-value (Significance)**	**Critical F-value**
Between Groups	0.38	1	0.38	95.06	1.03E-05	5.32
Within Groups	0.03	8	0.004			
Total	0.41	9				

*Error rate – 95 % confidence with an error rate of 5%

Table S5 ANOVA test of WEs and AgNPs against *E.coli*

SUMMARY						
Groups	**Count**	**Sum**		**Average**		**Variance**
WEs	5	3.90		0.78		0.0045
AgNPs	5	6.25		1.25		0.0075
ANOVA						
Source of Variation	**Sum of squares**	**Degrees of freedom**	**Mean square**	**F-statistic**	**P-value (Significance)**	**Critical F-value**
Between Groups	0.55	1	0.55	92.04	1.16E-05	5.32
Within Groups	0.05	8	0.01			
Total	0.60	9				

*Error rate – 95 % confidence with an error rate of 5%

Table S6 ANOVA test of AgNPs against *S.aureus* and *E.coli*

SUMMARY						
Groups	**Count**	**Sum**		**Average**		**Variance**
S. aureus	5	5.70		1.14		0.003
E. coli	5	6.25		1.25		0.0075
ANOVA						
Source of Variation	**Sum of squares**	**Degrees of freedom**	**Mean square**	**F-statistic**	**P-value (Significance)**	**Critical F-value**
Between Groups	0.03	1	0.03	5.76	0.043	5.32

Within Groups	0.04	8	0.01			
Total	0.07	9				

*Error rate – 95 % confidence with an error rate of 5%

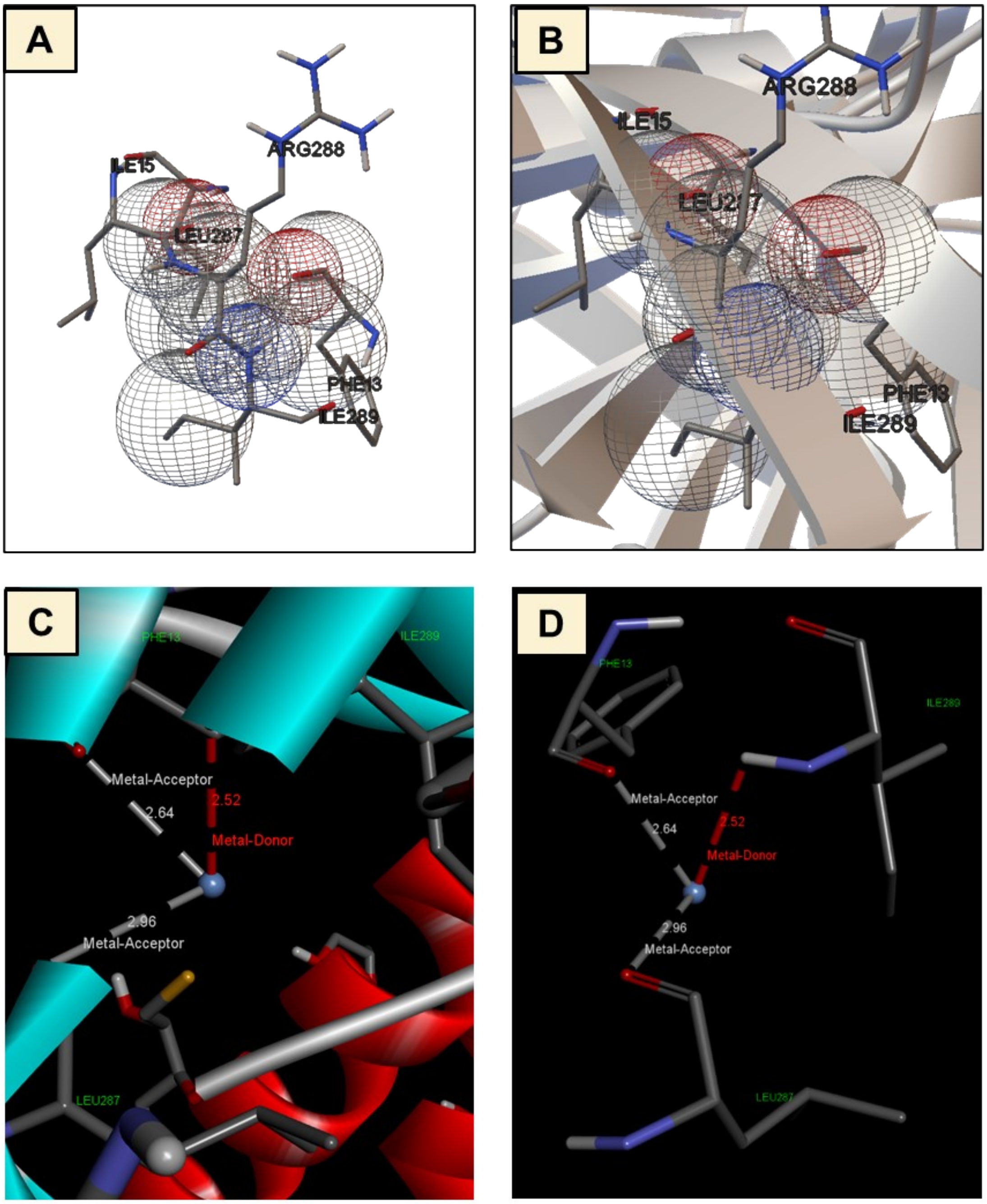

Figure S11. 3D interactions of Ag atom with protein Mre-11 viewed through (A-B)-AutoDock Tools and (C-D)-BIOVIA.

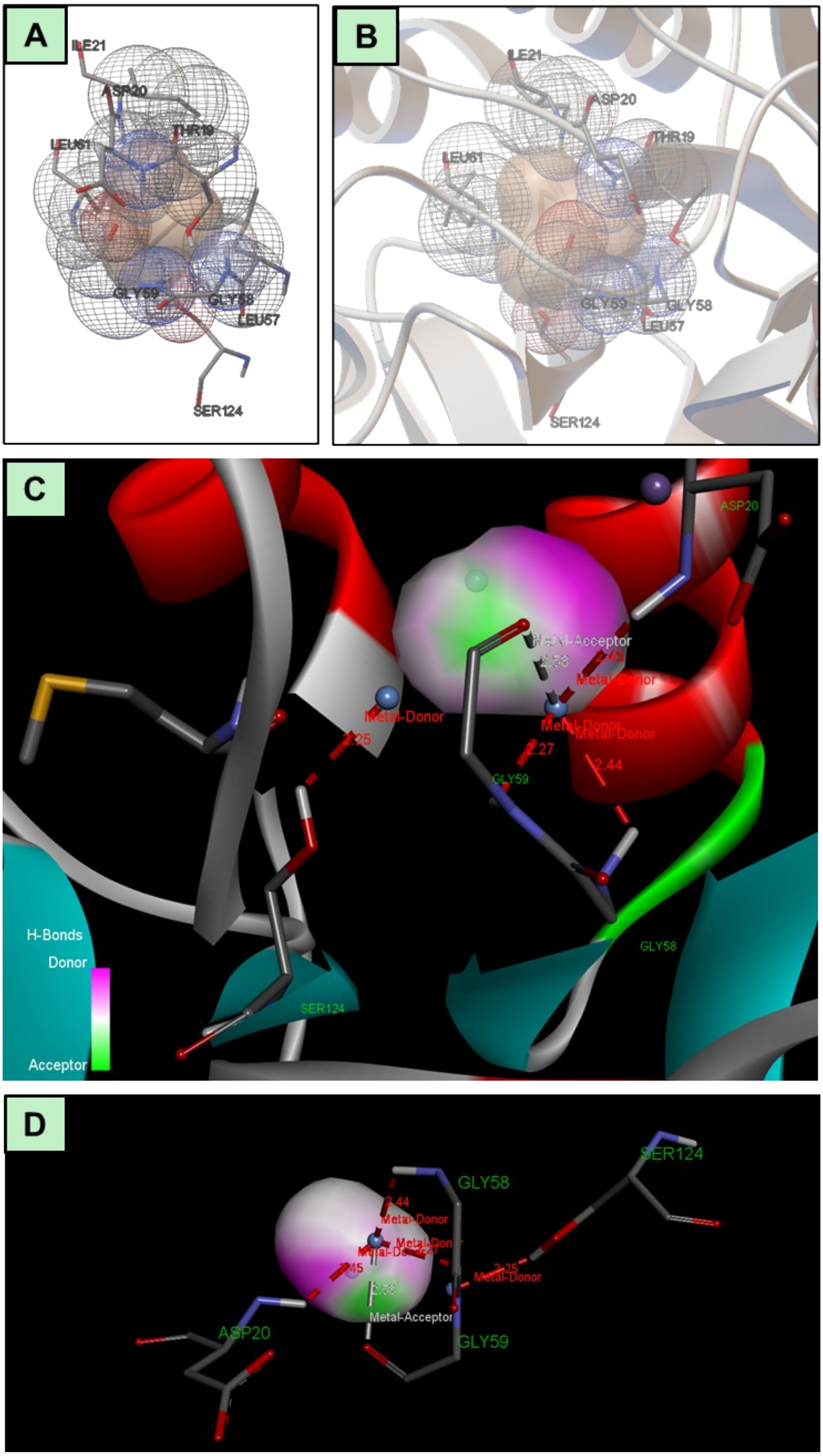

Figure S12. 3D interactions of Ag cluster with protein Mre-11 viewed through (A-B)-AutoDockTools and (C-D)-BIOVIA.
